# The History and Capabilities of Herbal Simples

**Published:** 1892-06-18

**Authors:** W. T. Fernie


### The History and Capabilities of Herbal Simples.

By W. T. Fernie, M.D.

*(Continued from page 288, Vol. XI.)*

### XXXIX.—THE FIG.

From time immemorial roasted figs split open, and applied hot against gumboils, and abscesses in other parts where a poultice would be inconvenient, have found widespread popular favour. The fruit, though indigenous to Western Asia, has been cultivated in most countries from a remote period, whilst growing wild in the warmer temperate regions of Southern Europe. "There is but one kind of fig," said Austin in 1665, "that comes to ripeness with us in England—the great blue fig, as large as a Catherine pear. This may be eaten with pepper and salt, and it nourishes exceedingly." Ages before then Dodonœus wrote about figs: "*Alimentum amplius quam cœteri prœbent,*" "they nourish much, and breed store of blood." In the green state they contain a milky, acrid juice, which will destroy warts, and which afterwards becomes saccharine. When ripe and dried they are emollient and laxative, affording a large percentage of glucose (sugar), with gum, fats, phosphates, and saline constituents. This sugar, being united with much mucilaginous matter of a smooth, oily nature, has long caused the figs of commerce to be esteemed as demulcent and pectoral. They are used in making the compound barleywater of the pharmacopœia, which also contains liquorice root and raisins; likewise in concocting the confection of senna. Two ounces of dried figs boiled in half a pint of water, and strained, will make a useful gargle for inflammatory sore throats. The fig is said to have been the first fruit eaten by man, and its leaves formed the most primitive dress of our progenitors in Paradise. Among the Greeks it made part of the Spartan daily diet. The Athenians set such store on the fruit that they forbade the best figs to be exported, and informers about those who broke this law were called "sukophantai," or fig discoverers—now known reproachfully by us as tale-bearing sycophants. Botanically, the fig is named ficus, as derived from the Greek word, "phuo," I generate. Bacchus is thought to have derived his corpulency and his vigour from this fruit; whilst Pliny tells us that figs were given of old to professed champions and wrestlers in order to strengthen and refresh them. He further says they are highly restorative, and the best thing for those to eat who are brought low by languishing disease, and have lost their colour. If added to ptisans for catarrhal affections of the air passages and other mucous canals, they act very beneficially. In the French codex, "*fruits pectoraux*" are composed of figs, stoned dates, jujubes, and raisins. When eaten by ourselves their skins and seeds act mechanically to prevent constipation. Beneath the peepul, or "*ficus religiosa,*" Buddha is supposed to have attained Nirvana. In the New Testament we are admonished that men do not gather grapes from thorns, or figs from thistles. Nevertheless, the fig tree belongs actually to the nettle tribe of plants, with innumerable little seeds within each so-called fruit, which is only a pulpy receptacle bearing the numberless tiny seeds or real fruits on its inner surface. This tree in the East was always green, and bore fruit several times in the year without observing any certain seasons; a fact which accounts for the Saviour visiting the fig tree by the road-side, "lest haply he might find fruit thereon," notwithstanding "the time of figs was not yet." The want of blossom on the fig tree was considered one of the most grievous calamities by the Jews; and in ancient times, it being found that most of the fruit often withered and dropped off before it became ripe, whilst the surviving figs which reached maturity were seen to have been perforated by certain winged insects, the process of ripening figs artificially by caprification was introduced. This was pursued by tying near the young figs some fruit of the wild fig tree, in which the said insects were known to abound, so that the flies should pass from the wild fruit and penetrate the cultivated figs at just the critical moment of their growth, when this fertilisation by insects was most opportune; and thus the effect of their eventually becoming ripe was secured. In this country we cannot raise figs from the ripe fruit of our trees, which always remain barren as seed. It is remarkable that fresh meat if hung for a few hours among the leaves of a fig-tree will become as tender as if kept elsewhere for weeks. Every butcher, therefore, ought to be able on occasion to "sit under his own fig-tree." Foreign figs are dried in an oven before importation, so as to destroy the larva of the gnat (a culex, or cynips), and are then compressed within small boxes, being of a yellowish brown colour, and filled with a viscid sweet pulp containing the dried achenes. The best Smyrna figs are branded with the Turkish word "Eleme," meaning choice, or selected. Domestic fowls have a perfect passion for figs after once tasting them. The fruit promotes suppuration if opened and applied to raw unhealthy sores. In the year 713 before Christ, when the King Hezekiah was sick unto death, the prophet Isaiah said "take a lump of figs," and they took it, and laid it on the boil, and he recovered. A sort of jam, to which the name figuine is given, is now prepared by our manufacturing grocers from the sweet pulp of the fruit for palatable use at breakfast as a natural aperient. Our fresh English fig when ripe is soft, easily digested, and corrective of strumous disease. The first fig-trees introduced into England (1540) are still alive and fruit-bearing in the Archbishop's garden at Lambeth, having been planted there by Cardinal Pole in the time of Henry the Eighth. Likewise a veteran fig-tree graces the Dean's garden at Winchester, trained against a stone wall, and bearing an inscription that in 1623 King James the First "tasted of its fruit with great pleasure." Gerarde says the fig-tree was cultivated here in 1597; but never cometh to kindly maturity unless it be planted under a hot wall and screened from the east wind. In the sixteenth and seventeenth centuries, the name "ficus," or fig, was given in England to a sore or scab growing about the body where hair is, or to a red sore in the fundament (Littleton, 1684). From this circumstance was derived the title of the Water Figwort (Scrophularia aquatica), which grows commonly by the sides of our ditches. This plant is called in some counties Brownwort, and in Yorkshire Bishop's Leaves, or *l'herbe du siège*—a term (*siège*) applied both to a latrine and to a bishop's see. It has been known from early times as an admirable vulnerary for fresh wounds, and its tubercled roots have been employed, on the doctrine of signatures, as an amulet against piles; likewise, an ointment is made from the leaves for the same painful affection. Respecting figs in classic days, a doggerel proverb obtained in the School of Salernum to the effect that "*Scrofa, tumor, glandes, Ficus cataplasma sedet*"—"Swine's evil, swellings, kernels, a plaster of figs will cure." We may suppose the fruit to have been esteemed as a luxury in the time of Shakespeare, who makes Charmian say, I love "long life better than figs"; but when he writes about "a fig for Peter" in *Henry VI.*, he clearly alludes, not to the fruit, but to the Spanish "*fico*—a mere snap of the fingers. *Faire la figue* is a general mode of insult in many parts of Europe, and consists in thrusting the thumb inserted between two closed fingers into the mouth. Our modern slang phrase, to "come out in full fig," owes its origin to the Italian *in fiocchi*, which means "in gala costume." Imitating the pompous style of Dr. Johnson, and ridiculing the solemn language employed by an Eastern fruit-seller in his most ordinary business, Horace and James Smith, in "Rejected Addresses," humorously describe such a street vendor as pretentiously hawking about, "In the name of the Prophet!! FIGS!!!"

